# *Bacillus licheniformis* FMCH001 Increases Water Use Efficiency via Growth Stimulation in Both Normal and Drought Conditions

**DOI:** 10.3389/fpls.2020.00297

**Published:** 2020-04-07

**Authors:** Saqib Saleem Akhtar, Daniel Buchvaldt Amby, Josefine Nymark Hegelund, Lorenzo Fimognari, Dominik K. Großkinsky, Jesper Cairo Westergaard, Renate Müller, Lars Moelbak, Fulai Liu, Thomas Roitsch

**Affiliations:** ^1^Department of Plant and Environmental Sciences, Faculty of Science, University of Copenhagen, Taastrup, Denmark; ^2^Plant Health Innovation, Chr-Hansen A/S, Hørsholm, Denmark; ^3^Department of Adaptive Biotechnologies, Global Change Research Institute, Czech Academy of Sciences, Brno, Czechia

**Keywords:** antioxidants, biostimulants, plant growth promoting rhizobacteria, plant probiotics, water use efficiency

## Abstract

Increasing agricultural losses due to biotic and abiotic stresses caused by climate change challenge food security worldwide. A promising strategy to sustain crop productivity under conditions of limited water availability is the use of plant growth promoting rhizobacteria (PGPR). Here, the effects of spore forming *Bacillus licheniformis* (FMCH001) on growth and physiology of maize (*Zea mays* L. cv. Ronaldinho) under well-watered and drought stressed conditions were investigated. Pot experiments were conducted in the automated high-throughput phenotyping platform PhenoLab and under greenhouse conditions. Results of the PhenoLab experiments showed that plants inoculated with *B. licheniformis* FMCH001 exhibited increased root dry weight (DW) and plant water use efficiency (WUE) compared to uninoculated plants. In greenhouse experiments, root and shoot DW significantly increased by more than 15% in inoculated plants compared to uninoculated control plants. Also, the WUE increased in FMCH001 plants up to 46% in both well-watered and drought stressed plants. Root and shoot activities of 11 carbohydrate and eight antioxidative enzymes were characterized in response to FMCH001 treatments. This showed a higher antioxidant activity of catalase (CAT) in roots of FMCH001 treated plants compared to uninoculated plants. The higher CAT activity was observed irrespective of the water regime. These findings show that seed coating with Gram positive spore forming *B. licheniformis* could be used as biostimulants for enhancing plant WUE under both normal and drought stress conditions.

## Introduction

Changing climatic conditions due to global warming pose severe environmental stresses to crops, which consequently affect their growth and yield. Among these stresses, drought is considered the single most devastating environmental stress, which decreases crop productivity more than any other environmental stress (Farooq et al., 2009). Drought disturbs water relations, reduces water use in plants, and subsequently impairs normal growth (Liu et al., 2005). In addition, drought stress also induces reactive oxygen species (ROS) such as superoxide radicals, hydrogen peroxide, and hydroxyl radicals resulting in oxidative stress (Noctor et al., 2014). At high concentrations, ROS can cause damage to various levels of organization, e.g., initiate lipid peroxidation, membrane deterioration, and degradation of proteins, lipids, and nucleic acids in plants (Sgherri et al., 2000; Hendry, 2008; Nair et al., 2008).

Various strategies have been suggested to improve the tolerance of plants to drought stress—such as traditional breeding and the genetic engineering of drought-tolerant transgenic plants (Farooq et al., 2009; Tardieu et al., 2018). Unfortunately, the results of these strategies are slow to implement in the field and require significant economic and technical investments.

One alternative approach is to use plant growth promoting rhizobacteria (PGPR) to enhance plant performance in dry growth conditions. PGPR are gaining importance as sustainable agricultural tools for integration into conventional agricultural practices. PGPR (such as *Bacillus* spp., *Pseudomonas* spp., and others) have been reported to confer resistance to various crops to biotic and abiotic stresses through a variety of mechanisms including direct change of the rhizosphere microbiota and/or manipulation of key plant metabolic pathways related to plant growth and stress responses (Nadeem et al., 2014; Naveed et al., 2014; Vejan et al., 2016; Vurukonda et al., 2016; Kumar and Verma, 2018).

Among the most promising PGPR, the Gram-positive spore forming *Bacillus* is gaining increasing attention due to its inherent stability and extended shelf life (Leser et al., 2008), making it ideal to be used in agricultural settings. PGPR spores are metabolically dormant and can resist very harsh environmental conditions such as heat, pH fluctuation, and desiccation (Setlow, 1994; Nicholson et al., 2000). *Bacillus* spores have a stability of more than two years, are easy to formulate and apply, do not germinate in tap water, and are not affected by conventional pesticides. These features allow *Bacillus* to be formulated together with most of the chemical additives that farmers and seed distributors normally employ for agricultural management practices as seed coating agents or in liquid media for in-furrow applications. The spores remain metabolically dormant until the presence of water and root exudates triggers spore germination and as a consequence shift to vegetative metabolically active cells. *Bacillus licheniformis* is a facultative anaerobic bacterium capable of anaerobic respiration and fermentative growth (Clements et al., 2002) which makes it suited to life in the rhizosphere due to the changing oxygen levels of drought/flooding periods. There is a high diversity within *Bacillus* which feature smaller genomes of about 4 MB but with the ability to produce a wide array of active compounds that are known to have antimicrobial and plant growth promoting activities and to induce plant defense (Ryu et al., 2004; Chen et al., 2007). *Bacillus* sp. confer resistance against both biotic and abiotic stresses in a variety of plants (Kumar and Verma, 2018). However, there is little information available on the use of such PGPR on improving plant water use efficiency (WUE) which is an essential parameter to evaluate drought resistance in plants. Traditional plant phenotyping methods are commonly used for monitoring plant drought responses. This can be via ecophysiological measurements which often include destructive harvest of plants for total biomass measurements or using various molecular and biochemical methods, which can be labor-intensive and time-consuming. Recently, multispectral imaging has been used for analyzing the growth of plants exposed to drought (Honsdorf et al., 2014). These image analyses could be combined with eco- and cell physiological methods to obtain a non-invasive characterization of plant performance (Großkinsky et al., 2015, 2018).

The present study was performed to understand the mechanisms of *B. licheniformis* FMCH001 in inducing drought resilience in maize by studying growth, ecophysiology, and metabolic changes during progressive drought and recovery.

## Materials and Methods

### Plant Material

Maize (*Zea mays* L.) cultivar Ronaldinho was grown in homogenized soil from a research field at Højbakkegård, Taastrup, Denmark (University of Copenhagen). The soil was classified as sandy loam, pH 7.2, total C 12.5 g kg^–1^, total N 1.4 g kg^–1^, water-soluble P 24 mg kg^–1^, exchangeable Ca 3.0 mmol kg^–1^, and exchangeable K, Mg, and Na < 1.0 mmol kg^–1^. Maize plants were grown in two different experimental setups, in an automated high-throughput phenotyping platform (PhenoLab) and under greenhouse conditions (University of Copenhagen, Taastrup).

### PhenoLab Experiments

**FIGURE 1:**
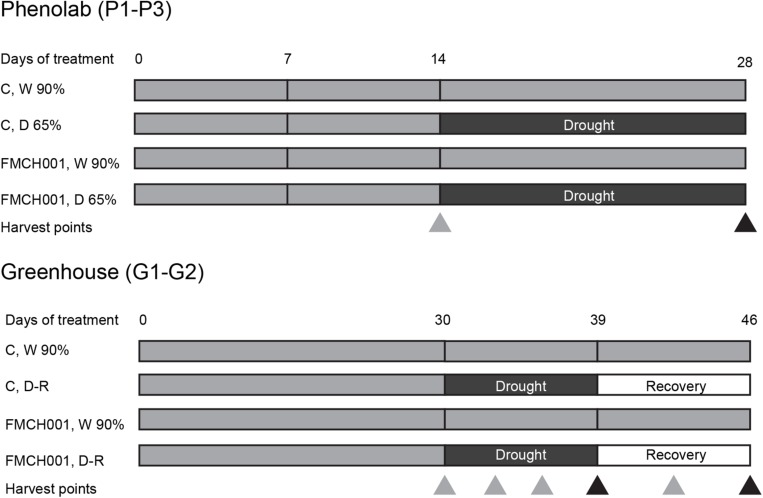
Schematic diagram showing the timeline of PhenoLab (P1–P3) and Greenhouse (G1–G2) experiments. Δ indicates destructive harvesting points. Black triangles denote harvest points in the end of treatments whereas gray triangles describe interim harvests. W 90% indicates well-watered treatment; D 65% indicates drought stress, D-R indicates the plants were exposed to drought stress and then re-watered. C indicates uninoculated control plants while FMCH001 indicates plants inoculated with seed coated *Bacillus licheniformis* sp. FMCH001.

For experiments in the automated greenhouse phenotyping facility PhenoLab described below (P1–P3), two mock- or *Bacillus licheniformis* FMCH001-coated seeds per pot were sown. Pots were 13 cm × 13 cm filled with 1.6 kg air-dried soil. Sowing depth was 3 cm. Germination and initial growth was done in greenhouse cells with 22°C day/16°C night regime for all PhenoLab experiments, but with no supplemented lights in P1 (summer: July and August) and P2 (autumn: September and October). P3 was carried out during winter (November and December) with natural light supplemented with an 18 h photoperiod of artificial light from high pressure sodium (HPS-SON-T 600W; E-Papillon, Netherlands) and LED (FL300 SUNLIGHT fixture from Fiona Lighting; Senmatic A/S, Denmark) lamps with a total intensity of 200 μmol m^–2^ s^–1^; 200 mL water was added per pot at the day of sowing and 2 days after sowing. Five days after sowing, plants emerged and 100 mL fertilizer solution containing macronutrients of 100 mg L^–1^ (Pioner NPK Makro 14-3-23 + Mg; pH 6.0, EC 2.0) per pot were added. Seedlings were carefully removed to obtain a single plant of comparable size and developmental stage per pot, facilitating uniformity of experimental plants. Seven days after sowing, 48 pots with one plant in each (24 mock- and *B. licheniformis* FMCH001-coated each) were inserted into the PhenoLab platform and automatically randomized with every watering and imaging session to measure crop coverage of individual plants (explained in detail below). The temperature and light conditions of the PhenoLab were similar to the greenhouse cell for seed germination, emergence, and initial growth stage. All plants were kept at well-watered conditions of 90% field capacity (FC) until day 14 using fertilizer solution, before half of the mock- and FMCH001-treated plants were exposed to drought stress (65% FC), while the other half was kept at 90% FC for another 14 days. To assure the same amount of nutrient supply to plants exposed to different irrigation and seed treatments, the fertilizer solution was stopped at day 14 and only tap water was added. To maintain the desired water contents, water was automatically supplied twice a day at the watering station (approximately 8 a.m. and 4 p.m.), when needed. Soil water content was determined by the difference of five soil samples of 150 g and their weights after drying to constant weight for 2 days at 70°C. Images for the determination of crop coverage were automatically captured two times daily for each individual plant in the PhenoLab platform (see below). Samples were taken at day 14 and 28 before and after drought treatments (Figure 1) to determine the above and below-ground dry weight (DW). The PhenoLab experiment was repeated three times (P1–P3).

### Greenhouse Experiments

The automated phenotyping facility PhenoLab was intended as a platform to pre-screen for non-destructive markers for beneficial effects of a bacterial strain using multispectral imaging for the early developmental stages of maize plants. In addition, to grow larger plants, regular greenhouse experiments with maize plants were conducted in plastic pots (diameter 15 cm; height 50 cm) with 10 kg of air-dried soil under same temperature and light conditions as for plants in PhenoLab experiments. Pots were arranged in a completely randomized design. To ensure a sufficient supply of nutrient during experiments, the recommended doses of N, P, and K (150, 380, and 130 mg kg^–1^ soil) were supplied as NH_4_NO_3_, KH_2_PO_4_, and K_2_SO_4_, respectively. Two mock- or FMCH001-coated seeds per pot were sown and the same selection criteria (uniformity and developmental stage) were made as in PhenoLab experiments (P1–P3). All plants were irrigated uniformly to maintain well-watered conditions (90% of pot water holding capacity) during the first 30 days of growth. After that, half of the pots were well watered, while the remaining half were subjected to progressive drought by withholding irrigation until the transpiration of the droughted plants decreased to 10% of the well-watered plants. Thereafter, drought-stressed plants were re-irrigated (recovery period) to the level of 90% pot water holding capacity for 7 days until the final harvest on day 46. Plant parameters were monitored during the experiment when samples were taken at days 30 (directly before drought was applied), 33, 36, 39 (3, 6, and 9 days of drought, respectively), 42, and 46 (3 and 7 days of drought recovery, respectively) (Figure 1). At each sampling day, flag leaves and roots of four plants per treatment were harvested. Roots were collected by carefully washing potted soil with a gentle stream of water in a sieve to avoid loss of detached root parts. These samples were snap frozen in liquid nitrogen and stored in −80°C for subsequent processing and analyses of carbohydrate and antioxidant enzymatic activities. At all sampling days, four plants per treatment were harvested for shoot and root biomass determination. The DW was determined after 48 h incubation at 70°C. In addition, physiological parameters as specified below were determined on the same days. Pots were arranged in a completely randomized design with four replicates of each treatment and the experiment was repeated twice (G1–G2).

### Automated High-Throughput Phenotyping

PhenoLab is a custom-made indoor phenotyping platform installed in a controlled greenhouse chamber at the University of Copenhagen, Denmark (Kuska et al., 2018). The platform consists of a conveyor system (ProInvent A/S, Hørsholm, Denmark), a watering station with the possibility of weight determination of pots (ProInvent A/S, Hørsholm, Denmark), and an advanced imaging station (Videometer A/S, Herlev, Denmark). A maximum of 117 fixtures can be inserted into the conveyor system, with 13 lanes of nine fixtures, which are connected by a circular conveyor transporting fixtures to and from the watering and imaging station. The setup allows for an automated randomization of fixtures. The setup of the watering station allows for top and bottom watering and can integrate up to four different water sources (reservoirs), e.g., for different nutrient supply. At the watering station, soil water content can be estimated by gravimetric determination or by deployed soil humidity sensors (Flower Power, Parrot Drones S.A.S., Paris, France), which are read out at the water station via Bluetooth. Irrigation for each fixture is adjusted according to the individual soil water content, thus allowing for defined drought treatments. The imaging station is equipped with a hemispheric image acquisition setup to facilitate homogenous, diffuse illumination provided by 10 high power LEDs of different wavelengths (365, 460, 525, 570, 645, 670, 700, 780, 890, and 970 nm). The multispectral images, which consist of the 10 respective bands with a spatial resolution of four megapixels, were acquired using the integrated Autolight setup and were re-adjusted every second day in consideration of plant growth. Based on these images, the parameter “crop coverage” was determined by using the provided VideometerLab software (Videometer A/S, Herlev, Denmark). Images are automatically segmented into “plant pixels” and “non-plant pixels,” and the derived ratio is used to express the “crop coverage” (Kuska et al., 2018).

### Bacterial Strain, Cultivation, and Seed Coating

The bacterial strain used was *B. licheniformis* sp. FMCH001 provided by Chr. Hansen A/S, Hørsholm, Denmark. Seed coating with *B. licheniformis* sp. FMCH001 was performed by FMC Agricultural Solutions, Hørsholm, Denmark by mixing 500 g of maize seeds (cv. Ronaldinio) with spray-dried FMCH001 and a sticking agent. The resulting seed coating had an average FMCH001 count of 2.5 × 10^6^ CFU per seed. CFU count was calculated by vortexing a coated seed in LB media and plating serial dilutions. For uninoculated control, seeds were coated with sticking agent.

### Leaf Gas Exchange Measurements

Leaf gas exchange, including photosynthetic rate (An), stomatal conductance, (gs) and transpiration rate (E), was measured from the upper canopy fully expanded leaves between 10:00 and 14:00 h with a portable photosynthetic system. Measurements were performed on 3 cm^2^ of leaf area at 400 μmol mL^–1^ of CO_2_ and 1500 μmol m^–2^ s^–1^ of photosynthetic active radiation (PAR) by a portable LI-6400 photosynthetic system (LI-COR 6400, Lincoln, NE, United States). Intrinsic WUE (WUEi) was calculated as the ratio of between An and gs and instantaneous WUE (WUE leaf) between An and E.

### Measurement of Leaf Water Potential

Total leaf water potential was measured with a pressure chamber (Soil Moisture Equipment Corp., Santa Barbara, CA, United States) on fully expanded upper canopy leaves between 10.00 and 12.00 h.

### Whole Plant Water Use Efficiency (WUEwp)

Water use efficiency at whole plant level was calculated as the ratio between the difference in the increase in shoot biomass (shoot DW at the end of each harvest-shoot DW at the beginning of drought) and the total water consumed by plants until that particular drought period.

Whole plant WUE was determined as follows:

$$W\,U\,E\,w\,p=\frac{\begin{array}{c} S\,h\,o\,o\,t\,D\,W\,a\,t\,t\,h\,e\,e\,n\,d\,o\,f\,e\,a\,c\,h\,h\,a\,r\,v\,e\,s\,t\\ -s\,h\,o\,o\,t\,D\,W\,a\,t\,t\,h\,e\,o\,n\,s\,e\,t\,o\,f\,d\,r\,o\,u\,g\,h\,t \end{array}}{\begin{array}{c} T\,o\,t\,a\,l\,w\,a\,t\,e\,r\,c\,o\,n\,s\,u\,m\,e\,d\,a\,t\,p\,a\,r\,t\,i\,c\,u\,l\,a\,r\\ h\,a\,r\,v\,e\,s\,t\,p\,o\,i\,n\,t\,d\,u\,r\,i\,n\,g\,d\,r\,o\,u\,g\,h\,t\,p\,e\,r\,i\,o\,d \end{array}}$$

Water consumption by plants was calculated by daily pot weighing during the drought period.

### Leaf Relative Water Content

Leaf relative water content (RWC) was determined according to Smart and Bingham (1974). In brief, leaf surface was cleaned with soft paper to remove any dust particle. Thereafter, small leaf discs were cut and leaf fresh weight (FW) was recorded. Then leaf discs were soaked in deionized water for 4–6 h and turgid weight (TW) was recorded. Thereafter, leaf discs are dried at 70°C for 48 h to record leaf DW.

Leaf RWC was calculated by using the following equation:

R⁢WC=⁢(FW-DW)(TW-DW)⁢X⁢ 100

### Enzymatic Activity Signatures of Carbohydrate and Antioxidant Metabolism

For the determination of enzyme activities of central carbohydrate metabolism and the antioxidative system, proteins were extracted according to Jammer et al. (2015). Extractions were done from flag leaves and roots of G1 and G2 at the end of drought and end of recovery. Briefly, 500 mg ground material was extracted with 1.5 mL extraction buffer (40 mM TRIS-HCl pH 7.6, 3 mM MgCl_2_, 1 mM EDTA, 0.1 mM PMSF, 1 mM benzamidine, 14 mM β-mercaptoethanol, 24 μM NADP). Cell wall-bound proteins were extracted from the remaining pellet with a high-salt buffer (1 M NaCl, 40 mM TRIS-HCl pH 7.6, 3 mM MgCl_2_, 1 mM EDTA, 0.1 mM PMSF, 1 mM benzamidine, 14 mM β-mercaptoethanol, 24 μM NADP).

The activities of the central carbohydrate metabolic enzymes cell wall, cytoplasmic and vacuolar invertases (cwInv, cytInv, vacInv; EC 3.2.1.26), fructokinase (FK; EC 2.7.1.4), hexokinase (HXK; EC 2.7.1.1), (fructose 1,6-bisphosphate) aldolase (Ald; EC 4.1.2.13), phosphoglucomutase (PGM; EC 5.4.2.2), phosphoglucoisomerase (PGI; EC 5.3.1.9), glucose-6-phosphate dehydrogenase (G6PDH; EC 1.1.1.49), ADP-glucose pyrophosphorylase (AGPase; EC 2.7.7.27), and phosphofructokinase (PFK; EC 2.7.1.11) were determined according to Jammer et al. (2015). FK, HXK, and PFK activities were below the level of detection in both roots and shoots (data not shown). Due to limited root material, invertase activities were measured only in shoots.

Activities of the antioxidant enzymes superoxide dismutase (SOD; EC:1.15.1.1), cell wall and cytoplasmic peroxidases (cwPOX, POX; EC:1.11.1.5), catalase (CAT; EC:1.11.1.6), ascorbate peroxidase (APX; EC:1.11.1.11), monodehydroascorbate reductase (MDHAR; EC:1.6.5.4), glutathione reductase (GR; EC:1.8.1.7), and dehydroascorbate reductase (DHAR; EC1:1.8.5.1) were determined according to Garcia-Lemos et al. (2019) and Fimognari et al. (2020).

All enzymatic activities were determined in a plate reader-based (BioTek Synergy 2) semi-high throughput approach in 96-well microtiter plates. The decrease or increase of substrate or product compounds (respectively) was monitored by the change in absorbance at the respective wavelength and the linear phase of compound conversion was used to calculate the enzyme activity in nkat g FW^–1^. For data evaluation, the Gen5 software (BioTek) was used. All assays were carried out in triplicates. Reactions with no substrate were used to estimate non-specific absorbance in extracts.

### Statistical Analysis

Data are presented as the means of eight replicates in PhenoLab (P1–P3) experiments and four replicates in greenhouse experiments (G1–G2) ± SE. Significance levels between or among treatments were determined at *P* < 0.05. Data from the PhenoLab experiments (P1–P3) and greenhouse experiments were analyzed by two-way anova (ANOVA) with Statistix ver. 8.1 software (Statistix, Tallahassee, FL, United States).

Statistical analysis of crop coverage was carried out by grouping the measurements within each series and assigning a common time stamp. Analyses were carried out using a linear mixed model with plant as random effect and the interaction between timestamp, water treatment, and FMCH001 treatment as fixed effect. An exponential correlation structure was included to capture the serial correlation in the plant specific curves using hours after initiation of experiment as the underlying timeline. Pairwise comparisons between treatments were made for all time stamps based on the estimated model and *p*-values were adjusted for simultaneous inference using the single-step approach proposed by Hothorn et al. (2008). The analyses were done by using the open-source statistical programming environment R version 3.4.2 (R Core Team, 2017) and in particular the packages nlme (Pinheiro et al., 2019) and multcomp (Hothorn et al., 2008).

## Results

### Screening for the Impact of *B. licheniformis* FMCH001 on Growth and Water Use Efficiency of Maize in the Automated Greenhouse Phenotyping Facility PhenoLab

In experiments P1 and P3, moderate drought regimes (65% FC) caused a reduction in shoot DW compared to plants grown under well-watered conditions (Table 1). FMCH001 treated plants showed an increase in shoot DW in P2 and P3 under well-watered conditions compared to control plants. In addition, root DWs of FMCH001 well-watered plants were higher than the uninoculated control in all PhenoLab experiments (P1–P3) but the significantly higher values of root DW were observed only in P1. Moreover, the root to shoot ratio was higher for well-watered plants with FMCH001 in P1 experiments compared to controls. Crop coverage estimated by image analyses of the plant canopy consistently showed non-significant higher values in FMCH001 treated plants in well-watered conditions compared to uninoculated control plants (Supplementary Figure S1). In addition, FMCH001 treated plants had comparatively higher values of WUE in P2 and P3 experiments when compared to the untreated controls under both well-watered and drought treatments (Table 1).

**TABLE 1:** Shoot dry weight, root dry weight, root/shoot ratio, and water use efficiency (WUE) of maize grown in PhenoLab experiments P1–P3.

	**Shoot dry weight (g)**	**Root dry weight (g)**	**Root/shoot ratio**	WUE (mg*mL^−1^)
	**P1**			
C, W 90%	5.30 ± 0.19a	1.51 ± 0.13b	0.29 ± 0.03c	6.32 ± 0.29a
FMCH001, W 90%	5.16 ± 0.20a	2.11 ± 0.15a	0.41 ± 0.02bc	7.08 ± 0.29a
C, D 65%	2.93 ± 0.31b	1.83 ± 0.18ab	0.64 ± 0.04a	6.98 ± 0.51a
FMCH001, D 65%	2.98 ± 0.24b	1.48 ± 0.07b	0.51 ± 0.04ab	6.67 ± 0.42a
	**P2**			
C, W 90%	1.19 ± 0.05a	0.29 ± 0.03a	0.24 ± 0.02 b	6.43 ± 0.18b
FMCH001, W 90%	1.34 ± 0.05a	0.34 ± 0.03a	0.26 ± 0.02ab	6.78 ± 0.22ab
C, D 65%	1.21 ± 0.04a	0.34 ± 0.03a	0.28 ± 0.02ab	7.25 ± 0.46ab
FMCH001, D 65%	1.19 ± 0.04a	0.38 ± 0.01a	0.32 ± 0.01a	7.49 ± 0.40a
	**P3**			
C, W 90%	3.20 ± 0.07b	1.20 ± 0.20b	0.38 ± 0.02ab	2.90 ± 0.13b
FMCH001, W 90%	3.87 ± 0.16a	1.22 ± 0.08b	0.31 ± 0.02b	3.32 ± 0.14a
C, D 65%	2.83 ± 0.14c	1.48 ± 0.17a	0.52 ± 0.05a	3.41 ± 0.17a
FMCH001, D 65%	2.88 ± 0.12c	1.50 ± 0.19a	0.52 ± 0.05a	3.48 ± 0.11a

*W 90% indicates well-watered treatment; D 65% indicates drought stress treatment; C indicates uninoculated control while FMCH001 indicates plants inoculated with seed coated *Bacillus licheniformis* sp. FMCH001. The values are means ± standard errors of the means (SE, *n* = 4). The different letters after the values in each column denote significant difference between the treatments at *P* < 0.05 level (Tukey’s test).*

### Characterization of the Impact of *B. licheniformis* FMCH001 on Maize in Large Pot Greenhouse Experiments

#### Physiological Response

To observe the effect of *B. licheniformis* FMCH001 on plant physiology, we recorded photosynthesis (An), stomatal conductance (gs), and calculated WUE at stomatal (WUE_i_) and at leaf level (WUE_leaf_). Data indicated no significant effects of FMCH001 inoculation on any of the leaf gas exchange parameters (An and gs) compared to uninoculated control plants under both normal and drought stressed conditions (Supplementary Table S1). Similarly, no effect of FMCH001 on plant WUE_i_ and WUE_leaf_ was observed compared to uninoculated control plants (Supplementary Table S1).

#### Growth Response

**FIGURE 2:**
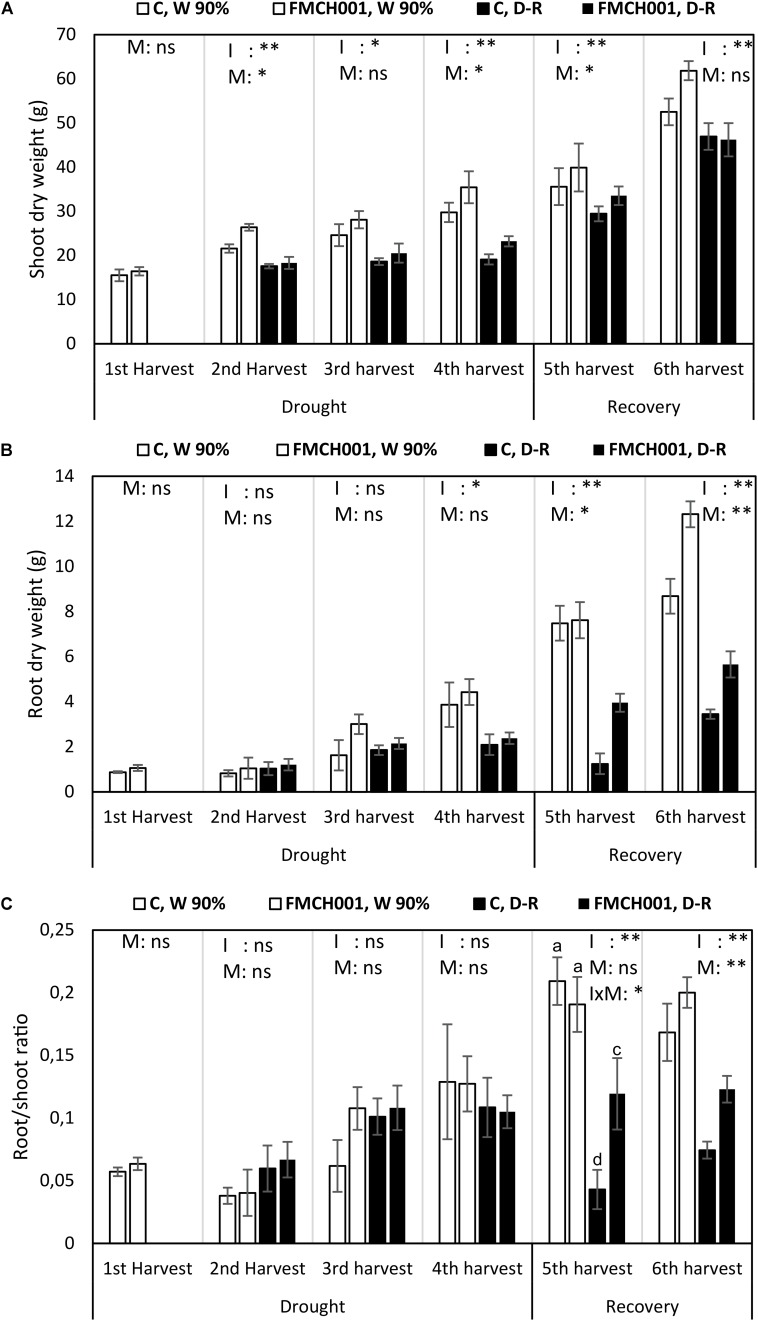
Shoot dry weight **(A)**, root dry weight **(B)**, and root/shoot ratio **(C)** of inoculated and uninoculated maize grown in Greenhouse experiments during drought and recovery period. W 90% indicates well-watered treatment; D-R indicates drought stressed plants which were re-watered during recovery. C indicates uninoculated control plants while FMCH001 indicates plants inoculated with seed coated *Bacillus licheniformis* sp. FMCH001. Bars represent mean ± SE (*n* = 4). M indicates microbial inoculation, I indicates drought treatment, and I × M indicates interaction between drought and microbial inoculation. The output of two-way ANOVA is also included where ^∗^ and ^∗∗^ denote significantly different at *P* < 0.05 and *P* < 0.01 levels, respectively, ns indicates no significant difference. Different letters on top of columns denote significant differences within the treatment at *P* < 0.05.

The growth stimulation effect of FMCH001 was studied under drought and during the recovery period (Figure 2). Drought stress drastically reduced plant growth at all harvests compared to the well-watered treatment.

Shoot DW of FMCH001 treated plants increased at all harvests under both well-watered and drought stress conditions when compared to uninoculated control (Figure 2A). At the second harvest, significantly higher (*P* < 0.05) shoot DW was observed in FMCH001 treated plants under well-watered conditions over uninoculated control. Whereas, at the fourth harvest (end of drought) FMCH001 treatment significantly increased shoot DW up to 16% in well-watered and 18% in droughted plants compared to uninoculated controls. In addition, at the fifth harvest, FMCH001 plants had more shoot DW than the uninoculated control indicating faster recovery of droughted plants treated with FMCH001.

The effect of FMCH001 treatment in maize was more pronounced on root growth compared to shoot growth. FMCH001 increased root DW under both well-watered and drought conditions. An increment in root DW of up to 46% at the third harvest under well-watered conditions and up to 68% at the fifth harvest in D-R plants with FMCH001 inoculation was observed, indicating faster root growth in inoculated plants than that of uninoculated control. In addition, significant effects of FMCH001 on root DW were noticed during the recovery period (re-watering), i.e., at the fifth and sixth harvest compared to the uninoculated control (Figure 2B).

Similar to root DW, FMCH001 significantly increased maize root/shoot ratio during the recovery period (i.e., at the fifth and sixth harvest) compared to the respective uninoculated control (Figure 2C). However, at the fifth harvest, the interaction between irrigation and microbes (*p* < 0.05) was also significant and statistically higher root/shoot ratio was observed in FMCH001, D-R plants.

#### Plant Water Relations

**FIGURE 3:**
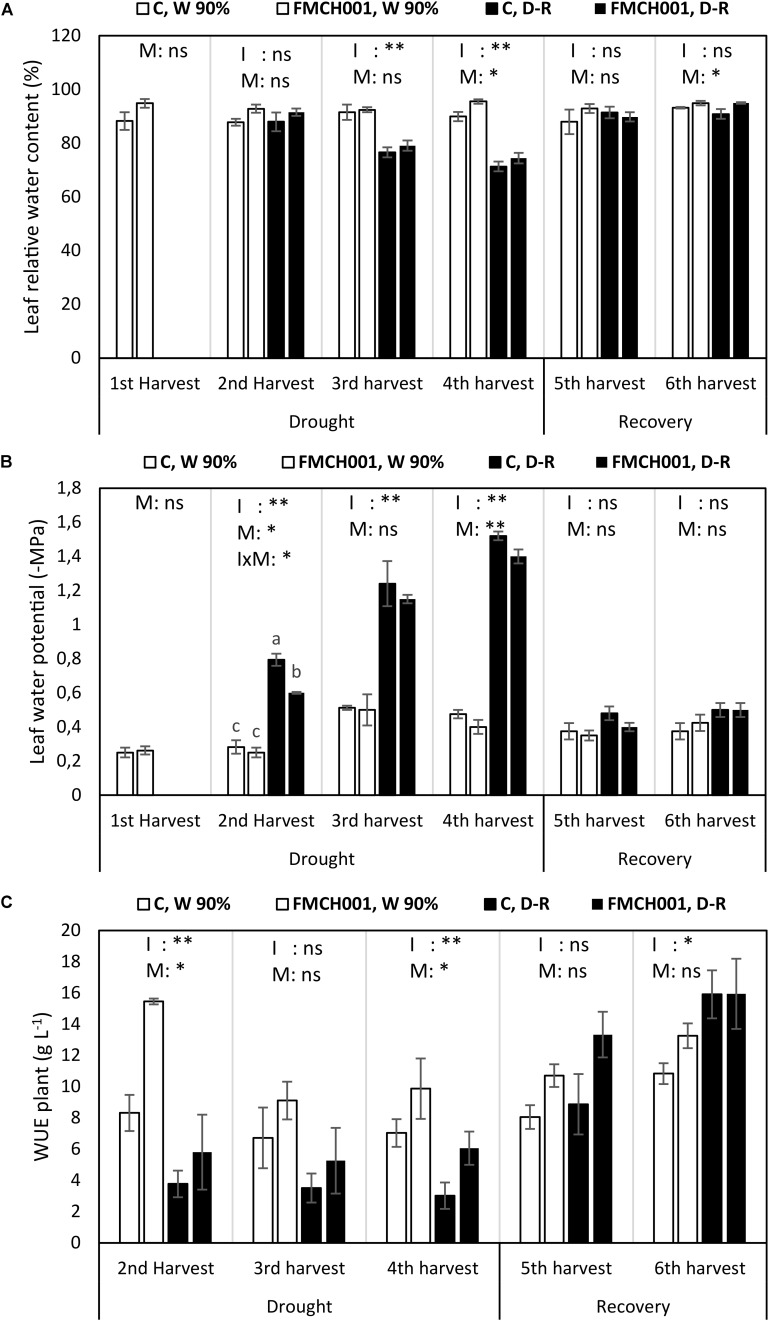
Leaf relative water content **(A)**, leaf water potential **(B)**, and plant water use efficiency **(C)** of inoculated and uninoculated maize grown in Greenhouse experiments during drought and recovery period. W 90% indicates well-watered treatment; D-R indicates drought stressed plants which were re-watered during recovery. C indicates uninoculated control plants while FMCH001 indicates plants inoculated with seed coated *Bacillus licheniformis* sp. FMCH001. Bars represent mean ± SE (*n* = 4). M indicates microbial inoculation, I indicates drought treatment, and I × M indicates interaction between drought and microbial inoculation. The output of two-way ANOVA is also included where ^∗^ and ^∗∗^ denote significantly different at *P* < 0.05 and *P* < 0.01 levels, respectively, ns indicates no significant difference. Different letters on top of columns denote significant differences within the treatment at *P* < 0.05.

FMCH001 increased maize leaf RWC at the fourth (end of drought) and at the sixth (last) harvest (Figure 3A).

Significant reduction in (more negative) mid-day leaf water potential (Ψ_leaf_) was seen with increasing drought progression. Under well-watered conditions, all plants maintained their Ψ_leaf_ regardless of the FMCH001 inoculation treatment. Whereas, drought stressed plants inoculated with FMCH001 showed comparatively higher Ψ_leaf_ (less negative) than that of uninoculated controls. In addition, Ψ_leaf_ was significantly higher (less negative) in FMCH001 drought stressed plants at the second and fourth harvest in relation to respective uninoculated control plants (Figure 3B).

The WUE at whole plant level (WUE_plant_) was higher in all FMCH001 treated plants than in the uninoculated controls regardless of irrigation treatment. However, the only statistically significant effect (*P* < 0.05) of FMCH001 was observed at the fourth harvest (Figure 3C).

#### Key Enzymes of Carbohydrate Metabolism and ROS Scavenging Enzymes Response

In the current study, 11 key enzymes of primary carbohydrate metabolism and eight ROS scavenging enzymes (antioxidants) were studied from root and shoot samples of maize. Data are presented in a heat map with color schemes ranging from red to green. For each enzyme, the maximum activity is presented with dark green color and the minimum with dark red color. Most antioxidant enzyme activities were found to increase in response to the drought treatment at the fourth harvest. However, of the studied enzymes, only CAT activity was found to respond in plants treated with FMCH001. The activity of CAT was consistently higher in roots (Table 2) but not in leaves (Supplementary Table S2) of plants treated with FMCH001 compared to the respective uninoculated control. Activities of the central carbohydrate metabolic enzymes did not respond to FMCH0001 inoculation when compared to the uninoculated controls.

**TABLE 2:** Root enzyme activity signatures of maize grown in Greenhouse experiment G1 under well-watered (W), and during drought and recovery period (D-R).

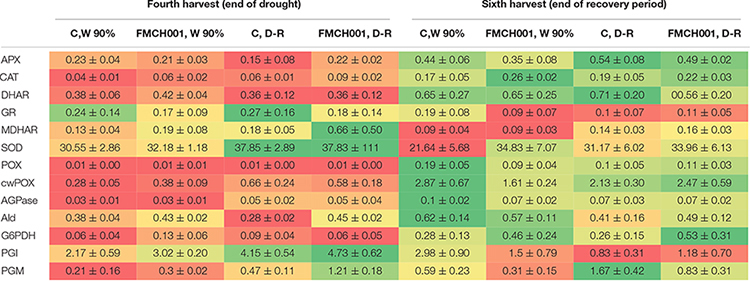

*W 90% indicates well-watered treatment; D-R indicates drought stressed plants which were re-watered during recovery; C indicates uninoculated control plants while FMCH001 indicates plants inoculated with seed coated *Bacillus licheniformis* sp. FMCH001. Values are means ± SE (*n* = 4). Data are presented as a heat map with color schemes for maximum enzyme activity (green) and minimum enzyme activity (red).*

## Discussion

Several PGPR have been reported to promote plant growth under drought stress through either direct or indirect mechanisms, or a combination of both (Gururani et al., 2013; Akhtar et al., 2015; Ngumbi and Kloepper, 2016). In several studies, PGPR belonging to the genus *Bacillus* offered advantages over other genera of PGPR in promoting plant growth under limited water conditions (Chakraborty et al., 2013; Kasim et al., 2013; Radhakrishnan et al., 2017). These bacteria stay as spores for their survival in water scarcity conditions, which help them to better survive under extreme conditions for longer periods compared to others. Additionally, these bacteria have been recognized as the most abundant in the root zone of drought-adapted plants. However, the effects of *Bacillus* on plant drought responses and in particular WUE remain to be studied. Furthermore, detailed physiological studies exploring the role of seed coated PGPR in plants in response to conditions of limited water availability are still lacking (Ma, 2019).

Here, we used the automated high throughput phenotyping screening facility PhenoLab that allows precise control of soil watering to decipher the potential of spore forming *B. licheniformis* FMCH001 in increasing drought tolerance in maize. The scenario of a couple of days of progressive drought severely affected maize growth. Plants inoculated with FMCH001 exhibited improved growth, which was observed as increased root DW, shoot DW, root/shoot ratio, and increased crop coverage in both well-watered and drought stressed plants compared to respective uninoculated control (Table 1 and Supplementary Figure S1). The crop coverage data based on multispectral images and increment in shoot and particular root biomass with FMCH001 in PhenoLab was further confirmed in big pot greenhouse experiments under both well-watered and drought stressed conditions (Figure 2). This is in accordance with Naveed et al. (2014) who reported that maize plants inoculated with *Burkholderia phytofirmans* strain PsJN had significantly higher root biomass (up to 70%) compared to uninoculated controls. Roots are considered to be one of the most important adaptive traits in enduring drought stress. Much evidence supports the fact that plants with a more prolific, deeper, and higher root biomass can tolerate drought stress better than plants with thinner root systems, as roots are the only organ capable of extracting water from the soil profile (Turner et al., 2001; Kavar et al., 2008; Gowda et al., 2011). In addition, increased root and shoot DW is directly related to plant WUE. Likewise, here in the greenhouse experiment, we found consistently higher plant WUE at whole plant level in FMCH001 treated plants compared to respective controls (Figure 2C). These results indicated that FMCH001 treated plants had better control of maintaining plant water status during progressive drought as represented by enhanced leaf RWC and less negative leaf water potential (Figures 3A,B) compared to uninoculated control plants.

Plant growth promoting rhizobacteria-mediated drought resistance has been studied extensively in plants (Lim and Kim, 2013; Akhtar et al., 2015; Calvo-Polanco et al., 2016; Ngumbi and Kloepper, 2016; Khan et al., 2018). The possible growth promotion mechanism might involve (i) production of plant growth promoting phytohormones by PGPR such as auxin, cytokinin, or ABA (Cassán et al., 2014; Castillo et al., 2015; Kumar et al., 2015; Maheshwari et al., 2015). (ii) Secretion of exopolysaccharide (EPS) which not only forms a biofilm/sheath around root surface to prevent it from desiccation stress but also involve binding soil particles resulting in an improved soil structure (Naseem and Bano, 2014; Nadeem et al., 2017; Niu et al., 2018). Very recently, Zheng et al. (2018) reported that soil inoculation with EPS producing *B. subtilis* can enhance soil water retention by reducing unsaturated soil hydraulic conductivity and by lowering soil evaporation rate compared to control. Hence, due to retaining more water in the soil for a longer period of time, EPS producing bacteria can enhance drought tolerance in plants either by providing more water to plants or by increasing the time available for metabolic adjustment for plants to better adapt to the drier condition. (iii) 1-Aminocyclopropane-1-carboxylic (ACC) acid deaminase activity of PGPR. ACC-deaminase may relieve plant stress particularly in drought conditions by degrading ACC into ammonia and a-ketobutyrate (Glick et al., 2007; Glick, 2012, 2014; Xu et al., 2014; Akhtar et al., 2015; Belimov et al., 2015; Saleem et al., 2015). Strain *B. licheniformis* is well known for its multi-functional traits such as auxin production, EPS secretion, and ACC-deaminase activity as reported earlier (Lim and Kim, 2013). Therefore, growth stimulation with FMCH001 under progressive drought and consequently faster recovery upon re-watering in the current experiment might be due to the multifunctional traits of the microbe.

An alternative explanation of growth promotion with *B. licheniformis* FMCH001 could be its role in the modulation of plant biochemistry under drought stress. The production of ROS such as H_2_O_2_ (hydrogen peroxide), O_2_^–^ (superoxide), and OH^–^ (hydroxyl) radicals in plant cells are well known under both normal and drought stress conditions. ROS play a critical role in plant development when present at low levels. However, their over-accumulation affects plant growth and development by producing an oxidation in the photosynthetic pigments, in membrane lipids, and in proteins and nucleic acids (Lushchak, 2014; Jajic et al., 2015). To regulate the level of ROS, plants produce antioxidants such as SOD, CAT, and APX. In the current study, we found that FMCH001 inoculated plants showed an increased activity of CAT in roots (Table 2). CAT neutralizes the negative effect of ROS by hydrolyzing H_2_O_2_ to water and oxygen. Hence, FMCH001 inoculated plants offered advantages over uninoculated control plants in regulating the level of ROS in plant cells. Similarly, Zhou et al. (2017) reported enhanced activity of SOD, CAT, APX, and GPX in Chrysanthemum inoculated with *B. licheniformis* SA03 under saline-alkaline conditions. In addition, very recently Chiappero et al. (2019) also reported similar findings, i.e., higher antioxidant capacity in inoculated plants in relation to uninoculated controls under drought stress.

It has been shown that modulation of carbohydrate metabolism in invertase-overexpressing tomato improves drought and salt tolerance, which was accompanied by changes in antioxidant metabolism (Albacete et al., 2014a, b). In contrast, the cell physiological analyses showed that only CAT was found to respond significantly to the FMCH001 inoculation whereas neither source nor sink metabolism was neither positively nor negatively affected as assessed via the determination of various cell and ecophysiological parameters. However, other work has shown that even effective physiological changes may be subtle and therefore difficult to capture (de Lima et al., 2019). Thus, our findings do not rule out regulation via subtle changes in specific temporal and spatial dynamics.

## Conclusion

*Bacillus licheniformis* FMCH001 applied as a seed coating to maize enhances plant WUE by producing more biomass (particularly root) which might be due to upregulation of antioxidative enzyme (CAT) under both well-watered and drought stress conditions. Hence, *B. licheniformis* FMCH001 could potentially be used as a biostimulant for enhancing crop productivity under varying environmental conditions. One of the greatest challenges facing humanity is to secure sufficient and healthy food for the increasing world population (Ehrlich and Harte, 2015). This requires maintaining the sustainable cultivation of crop plants under changing climate conditions (FAO and ITPS, 2015). The relationship between plant roots and soil microbes has existed since the emergence of plants on land (Delaux et al., 2015). Thus, the use of beneficial microbes such as *B. licheniformis* FMCH001 is a promising approach to improve crop resilience. However, the findings of these controlled environment experiments will need to be verified in field conditions to further confirm the growth stimulation effect of *B. licheniformis* FMCH001 on maize for practical applications in agriculture.

## Data Availability Statement

All datasets generated for this study are included in the article/Supplementary Material.

## Author Contributions

LM, FL, and TR conceived and designed the research. SA and DA planned and performed experiments with assistance of JH, DG, and JW. SA, DA, JH, JW, FL, and TR analyzed the data. SA and DA wrote the manuscript. JH and DG critically revised the manuscript. LF, RM, LM, FL, and TR provided expertise and feedback.

## Conflict of Interest

The authors LF and LM are employed by Plant Health Innovation, Chr-Hansen A/S.

The remaining authors declare that the research was conducted in the absence of any commercial or financial relationships that could be construed as a potential conflict of interest.
