# Supplementary material for: Bacillus licheniformis FMCH001 Increases Water Use Efficiency via Growth Stimulation in Both Normal and Drought Conditions
Source: Front Plant Sci. 2020 Apr 7;11:297. doi: 10.3389/fpls.2020.00297 (PMC7155768; doi:10.3389/fpls.2020.00297)
Supplement: Supplementary file 1 [file Image_1.pdf]

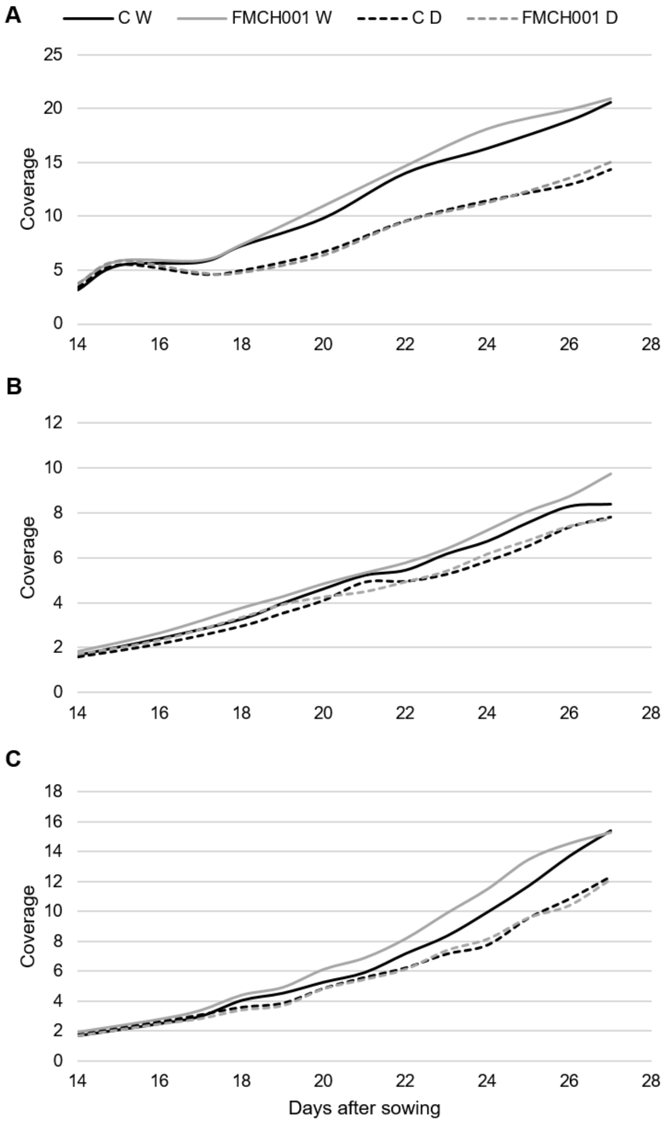

Supplementary Figure 1 Crop coverage of maize grown in PhenoLab experiments. Crop coverage of maize plants grown in PhenoLab experiments P1 (**A**), P2 (**B**) and P3 (**C**) were calculated based on image analyses. Plants were grown in well-watered (W, 90% FC) or drought (D, 65% FC) conditions. Seed coating were done using *B. licheniformis* FMCH001 (FMCH001) or for controls sticking agent with no bacteria (C). Experiments were conducted using eight replicates. Statistical analysis to compare measurements within a series did not result in any significant results. Statistical analyses were done in the open-source statistical programming environment R version 3.4.2 [1] and in particular the packages multcomp [2] and nlme [3].

[1] R Core Team (2017). R: A language and environment for statistical computing. R Foundation for Statistical Computing, Vienna, Austria. [2] Hothorn T, Bretz F and Westfall P (2008). Simultaneous Inference in General Parametric Models. Biometrical Journal: 50(3), 346-363. [3] Pinheiro J, Bates D, DebRoy S, Sarkar D and R Core Team (2015). nlme: Linear and Nonlinear Mixed Effects Models.
