# Supplementary material for: Bacillus licheniformis FMCH001 Increases Water Use Efficiency via Growth Stimulation in Both Normal and Drought Conditions
Source: Front Plant Sci. 2020 Apr 7;11:297. doi: 10.3389/fpls.2020.00297 (PMC7155768; doi:10.3389/fpls.2020.00297)
Supplement: Supplementary file 2 [file Table_1.pdf]

Supplementary Table 1 Leaf gas exchange parameters (photosynthetic rate (A_n), stomatal conductance (g_s)), and water use efficiency at stomatal (WUE_i) and at leaf level (WUE_{leaf}) of inoculated and uninoculated maize grown in Greenhouse experiments during drought and recovery from drought. W 90% indicates well-watered treatment, D-R indicates the plants were drought treated and then re-watered. C indicates uninoculated control while FMCH001 indicates plants inoculated with seed coated *Bacillus licheniformis* sp. FMCH001. Values are means ± SE. The output of two-way ANOVA is presented, where M indicates microbial inoculation, I indicates drought treatment, I x M indicates interaction between drought and microbial inoculation.

**Attributes**	**A_n**	**g_s**	**WUE_i**	**WUE_{leaf}**
		Drought period		
C, W 90%	28.93±1.19	0.24±0.01	124.40±3.53	6.26±0.16
FMCH001, W 90%	29.46±1.21	0.20±0.02	124.15±9.11	6.22±0.39
C, D-R	9.46±1.05	0.09±0.01	61.79±33.02	4.02±0.13
FMCH001, D-R	11.42±1.69	0.13±0.02	76.25±36.87	4.46±0.60
P values				
I	<0.001	<0.001	<0.001	0.069
M	0.415	0.445	0.768	0.633
I x M	0.634	0.175	0.812	0.565
		Recovery period		
C, W 90%	19.98±1.33	0.13±0.01	146.04±7.40	7.32±0.24
FMCH001, W 90%	16.88±2.86	0.10±0.02	163.94±5.30	7.85±0.30
C, D-R	22.54±1.46	0.14±0.01	151.46±3.88	7.76±0.29
FMCH001, D-R	17.71±0.90	0.12±0.00	135.39±8.16	7.07±0.16
P values				
I	0.328	0.182	0.147	0.489
M	0.038	0.108	0.903	0.726
I x M	0.609	0.594	0.045	0.028
