# Supplementary material for: Bacillus licheniformis FMCH001 Increases Water Use Efficiency via Growth Stimulation in Both Normal and Drought Conditions
Source: Front Plant Sci. 2020 Apr 7;11:297. doi: 10.3389/fpls.2020.00297 (PMC7155768; doi:10.3389/fpls.2020.00297)
Supplement: Supplementary file 3 [file Table_2.pdf]

Supplementary Table 2 Shoot enzyme activity signatures of maize grown in Greenhouse experiment G1 under well-watered (W) and during drought and recovery (D-R) period.

	4th harvest (end of drought)				6th harvest (end of recovery period)			
	C, W 90%	FMCH001, W 90%	C, D-R	FMCH001, D-R	C, W 90%	FMCH001, W 90%	C, D-R	FMCH001, D-R
APX	0.44±0.02	0.46±0.03	0.52±0.02	0.55±0.03	0.31±0.08	0.34±0.02	0.25±0.05	0.29±0.03
CAT	0.19±0.03	0.25±0.03	0.51±0.13	0.43±0.06	0.23±0.09	0.28±0.08	0.15±0.03	0.21±0.11
DHAR	0.20±0.01	0.24±0.07	0.23±0.03	0.17±0.04	0.22±0.03	0.26±0.03	0.26±0.04	0.24±0.02
GR	1.15±0.38	1.26±0.31	2.02±0.35	2.79±0.38	1.84±0.45	2.01±0.24	2.15±0.39	2.35±0.34
MDHAR	0.48±0.02	0.47±0.05	0.52±0.01	0.41±0.07	0.13±0.04	0.24±0.08	0.23±0.08	0.31±0.12
SOD	12.94±1.77	13.29±2.39	7.83±3.46	14.79±0.63	8.28±1.65	10.63±2.54	13.09±1.52	15.02±4.19
POX	1.61±0.24	2.02±0.48	2.14±0.43	1.96±0.41	2.24±0.37	1.95±0.32	1.76±0.36	2.54±0.33
cwPOX	0.65±0.53	1.56±0.53	1.53±0.72	1.87±0.55	1.78±0.92	1.27±0.48	1.23±0.56	1.63±0.17
AGPase	0.04±0.02	0.02±0.00	0.06±0.02	0.05±0.01	0.04±0.01	0.04±0.01	0.04±0.01	0.04±0.01
Ald	1.29±0.83	1.89±0.45	6.90±0.81	7.54±0.56	4.55±1.17	4.36±0.77	3.32±0.78	4.71±1.37
G6PDH	0.33±0.07	0.57±0.12	0.51±0.17	0.71±0.30	0.99±0.29	0.95±0.22	1.04±0.09	0.74±0.17
PGI	14.40±1.75	13.22±0.77	13.66±1.05	16.84±1.87	9.66±1.38	11.50±1.47	12.54±0.83	11.42±1.03
PGM	14.41±3.20	16.32±0.91	20.13±4.29	20.32±1.10	11.75±2.36	13.72±2.391	9.01±1.66	10.11±1.46
cwInv	4.68±1.51	4.23±0.96	4.53±1.50	5.45±0.86	6.60±1.65	8.20±1.54	8.45±1.60	7.25±1.79
cytInv	6.01±1.16	9.00±3.64	16.09±3.50	9.71±3.44	3.70±0.69	4.44±0.32	4.67±1.40	3.14±0.35
vacInv	8.26±1.18	8.85±0.087	39.29±3.39	30.51±5.41	8.26±1.65	8.26±1.54	8.26±1.60	8.26±1.79

W 90% indicates well-watered treatment; D-R indicates drought stressed plants, which were re-watered during recovery. C indicates uninoculated control while FMCH001 indicates plants inoculated with seed coated *Bacillus licheniformis* sp. FMCH001. Values are means ± SE (n=4). Data are presented as a heat map with colors schemes for maximum enzyme activity (green) and minimum enzyme activity (red).
